# He That Bloweth Not His Own Horn, the Same Will Not Be Tooted

**Published:** 1889-11

**Authors:** 


### "He that Bloweth not His own Horn the Same Will not Be Tooted."

We have placed the above heading in quotation marks because it is not original with us. We copied it from the *Southern Practitioner*, of Nashville. We subscribe, however, to the sentiment contained in it.

There are a number of methods of "tooting" one's self. When a doctor reports a case in a medical journal the "toot" he gives himself thereby is considered legitimate; but when he reports a case in a newspaper, or has it reported for him by a reporter, the "toot" is regarded as illegitimate.

The "toot" in a medical journal is largely legitimate, we presume, because when a physician selects that organ he must necessarily limit his reports to facts or he will be very apt to be exposed, for all the readers of medical journals are medical men, versed in diseases, and can not very well be imposed upon; and then it is expected that his case will be of a kind that will bring to the attention of the readers of the journal something new and instructive, by means of which they will be benefitted. Thus a person can be borne with in "tooting himself" if, when he does so, he increases knowledge and benefits humanity.

When a doctor "toots" himself in a newspaper, by having a reporter report a wonderful operation that he has performed, we give but little credence to any of the circumstances related. We conclude that the report has been manufactured for the sole purpose of "tooting," to get patients, of which he has probably a great dearth in his practice. On the day we are writing, we read a report in a daily newspaper, of Cincinnati, illustrating the remarkable acumen displayed by a certain medical gentleman in diagnosis. A child of a family, it was stated, some time ago had gotten one of its lower limbs badly injured. One physician after another, of the "highest standing," was called to attend to it. Each one pronounced that it had disease of the hip-joint, and stated there was no cure for it—it would necessarily be a cripple for life. Finally, after a lapse of over six months, the gentleman who displayed such great acumen was called in. At once he discovered that the trouble with the limb was not hip-joint disease, but a dislocation of the femur downward. He began manipulating the limb, and soon there was heard a report like the explosion of a pistol. So loud was the report that it was heard in a distant room. The cure was complete. What a boon he was to that poor child! was the declaration of every one.

But seriously, though it be granted that every statement of the report is true, what benefit would result from the report beyond the "tooting" of the doctor? No new or more accurate means of detecting dislocations at the hip-joint were developed. If the report had shown that the doctor by extensive research and hard study had discovered a simpler method than already known how dislocations could be diagnosed, we might have partially overlooked his selecting a newspaper instead of a medical journal for making it known to the public, but as such was not the case, we must have additional proof to that contained in the report before we can believe that he was so much *smarter* than his contemporaries.

The present time is not favorable for even laymen to believe in the "toots" of doctors when there is no other purpose subserved than the "tooting" of themselves.

When a surgeon amputates a limb, extracts a tooth, opens an abscess, removes an eyeball, why report the fact in a newspaper?

Thousands of limbs are amputated in and out of hospitals every day—so, the world over, millions of teeth are extracted every day. Supposing all the surgical operations performed every day in Cincinnati alone were published in the newspapers of this city; the result would be the papers would be filled by them to the exclusion of other reading.—*Medical News, Cin.*

---

COVER GLASSES, as sold nowadays, are not quite clean. They should be washed in distilled water and kept in a wide-mouthed bottle filled with alcohol acidulated with hydrochloric acid. They are readily cleaned with tissue or Japanese napkin paper between the thumb and forefinger. The patent devices for cleaning cover glasses are only serviceable to make a show of those who use them.

